# The Brown-Sequard Fluide Testiculaire

**Published:** 1889-09

**Authors:** 


BUFFALO MEDICAL AND SURGICAL JOURNAL

A MONTHLY REVIEW OF MEDICINE AND SURGERY.

*EDITORS:*

THOS. LOTHROP, M. D. - - - W. W. POTTER, M. D.

All communications, whether of a literary or business character, should be addressed to the editors: 284 FRANKLIN STREET, BUFFALO, N. Y.

### Editorial.

### THE BROWN-SÉQUARD FLUIDE TESTICULAIRE.

IN THE July number of the *American Journal of Insanity* appears a letter from Dr. J. B. Andrews, from which we extract that portion referring to Prof. Brown-Sêquard and his fluide testiculaire:

My next visit was made to Prof. Brown-Séquard, who has been so long and favorably known to the profession in America for his physiological researches, from his being the physician to the late Senator Sumner, from his lectures delivered in New York, now eleven years since, and from his published writings. I called upon him during the hour of one of his reception days, and was immediately ushered into his office. I found him feeling much stronger and better in health, as the result of some recent experiments made upon himself. He began at once to speak of his latest work, and the rejuvenating effects experienced by him. From his statement one might fairly conclude he had finally discovered the fountain of youth, but as he has already placed his experiments before the medical world in the last number of the *Gazette hebdomadaire*, I send you a translation made for me by Dr. Sherwood Dunn, formerly of New York, but now a graduate of the Paris Faculty of Medicine, and practising in that city. I know that your readers will be interested in a discovery which affects both the physical and mental life of man, removes all the deterioration of age, and reinstates the vigor of full manhood.

"Prof. Brown-Séquard made a verbal communication to the Society of Biology, of Paris, Saturday, June 1st, describing the remarkable effects which he had observed upon himself, following the subcutaneous injection of a liquid obtained by crushing the fresh testicles of guinea-pigs and dogs, with the addition of a small quantity of water.

The effects were such, that for himself he does not hesitate to declare that they were equivalent to restoring to him the vigor of several years. It is important to state that the savant president of the Society of Biology is seventy-two years of age, having been born April 8, 1817. During the past ten years his general vigor has notably diminished. His strength permitted of his remaining standing only a half-hour daily at his laboratory work; and he became exhausted after directing it for three or four hours in a seated position.

To the great astonishment of his assistants, the day following the second injection of the liquid, he could remain on his feet, at his work, for even three and one-quarter hours, without feeling a degree of fatigue that would cause him to sit down.

Other proofs of augmented vigor are given by the celebrated Professor. The bladder and the large intestine have notably gained in strength. The jet of urine, carefully measured after breakfast, during about ten days preceding the first injection› was inferior in distance, to the point of striking the walls of the water-closet cuvette, by about one-quarter of what it has become after the two first injections.

We need not say that these experiments have been made in conditions that assure their value, by similarity in food and drink, as well as in quantity and character. But the intestine has furnished a still greater proof to the learned experimenter. For many years he has been obliged, like many people of advanced age, to give mechanical aid to the action of the rectum. He no longer needs this assistance, even in the expulsion of matters much larger in size than has been his habit.

By the dynamometer he has also found an incontestible augmentation in the strength of his limbs, the forearm in particular. The average trials after the first two injections are superior, by six or seven kilogrammes, to those made before.

Though he is now subjected to greater causes of fatigue in his laboratory than formerly, he does not feel the necessity, as has been his constant habit for ten years past, to retire to bed immediately after the meal, hastily taken, upon returning from his experimental labors. Moreover, he affirms that intellectual work has become easier to him, and that he has regained, in this respect, all that he had lost for a number of years past. Also, he has noticed a marked augmentation of forces, which, although not lost, were sensibly diminished. These remarkable effects have been obtained, as we have said, by employing a process which we will describe after having noted how he was led to make these experiments.

We all know that eunuchs, or at least those who, in childhood, have been deprived of their testicles by ablation of these organs, and not by crushing, are weak mentally, morally, and physically. It is known, also, that a characteristic weakness exists in men, even young and naturally vigorous, who abuse the sexual power. These facts and some others have led Dr. Brown-Séquard to believe, and to teach, as he did in his lectures, in the School of Medicine, in 1869, that if it were possible to inject sperm, without danger, into the veins of old men, one could obtain in these manifestations of renewed youth. Guided by this idea, he made experiments in 1875 upon a dozen of old dogs, and tried in vain, with one exception, the injection of the sperm of guinea-pigs alone, and of the sperm of this animal mixed with that of the dog. The success obtained in the one case confirmed the views of the Professor, but the experimental processes were not such as could be used upon man. A few years since there occurred to the mind of the learned Professor another mode of application, and this is the one he lately employed upon himself. It consists in placing a ligature around the vasculo-nervous hilum of the testicle of a guinea-pig or dog, and, after having cut off this hilum above the ligature, one extirpates the whole testicle. The mass thus extracted is crushed, gland, blood-vessels and membranes together, from two to five cubic centimetres of distilled water are added, and the whole is thrown on a filter. Of the liquid thus obtained, part is employed immediately in a subcutaneous injection, and the remainder preserved in a vessel surrounded by ice, for subsequent injections. At the date of this article, eight of these injections have been made, six upon the lower members, and two upon the left forearm. These injections were made upon the 15th, 16th, 17th, 24th, 29th and 30th of May last. The average quantity of liquid employed for an injection being about one cubic centimetre. The three first injections were made with the liquid obtained from a dog; the others with the liquid coming from several young or adult guinea-pigs.

It seems certain that the liquid obtained from the dog's testicles was more efficacious than that of guinea-pigs. Nevertheless, it is on the day following the use of liquid obtained from a very young guinea-pig that Prof. Brown-Séquard found the maximum of favorable results.

We need not say that, before making these experiments upon himself, the learned Professor tried them numerous times upon animals, chiefly to make sure there was no danger in the injection of this special liquid under the skin. Its innocuous properties being satisfactorily demonstrated, the experimenter thought he could, with impunity, proceed with the injection upon himself. He was mistaken in some regards. Five of these injections, out of eight, gave him prolonged and intense pain (from five to twelve and fifteen hours), and an erythematous inflammatory swelling. Two of the points of injection are still painful; one five and one ten days after the operation.

Prof. Brown-Séquard ends his communication with the remark that the effects produced upon himself may possibly be attributed by the reader to his imagination; and he hopes that other physiologists will repeat his experiments, and demonstrate whether or not these effects are to be attributed to a special idiosyncrasy, or to a sort of suggestion without hypnotisation, augmenting the vigor of the nervous centers, and more especially the cord, or if it is due, as he thinks, to the influence of the fluid injected.

Many particulars are to be studied in order to resolve the great question attached to these interesting experiments, and Dr. Brown-Séquard will make them the subject of further communication to the Society of Biology."—*Gazette hebdomadaire*, June 7, 1889.

The Professor was so occupied in telling us of his supposed discovery that none of the time we felt at liberty to claim from him could be given to converse upon general subjects. I left him, thoroughly impressed by his genial manners and his kindness of heart. He is seventy-two years of age, has an appearance of more than ordinary vigor, is a constant worker in his laboratory, and retains a position in a school which demands annually an exhibit of original investigation as the *sine qua non* of its continuance.

I do not feel that, in writing this letter, I am doing justice either to your readers or to myself. You may, however, consider it as an introduction to the article concerning Prof. Brown-Séquard which accompanies it.

This will give our readers the most important information obtainable upon this subject up to the present.

We notice that Dr. Andrews is careful not to express his opinion concerning the injection of the fluide testiculaire.

Time and experience alone will tell whether the learned Professor has been deceived or not. A few isolated cases are not of sufficient weight to convince. By all means, let the experiments be multiplied, and the reports be published.

We hope that care will be taken in choosing the animal whose testicles are to furnish the vital force, for, should the injection have the latent power of transmitting the animal characteristics, we should have pugnacious old men rejuvenated by extract of bull-dog, playful old bald-heads indebted to some spaniel. The weasel might be utilized for producing watchfulness in aged detectives, for who ever caught a weasel asleep.

The possibilities of this injection are great, its probabilities small, but we should by no means pre-judge any announcement from so eminent a source.

